# Atomistic Simulations of Individual Amphiphilic Carbosilane Dendrimers with –(OCH_2_CH_2_)_n_–OCH_3_ Terminal Groups in Hydrophilic and Hydrophobic Environments and at Interfaces

**DOI:** 10.3390/polym18010092

**Published:** 2025-12-28

**Authors:** Andrey O. Kurbatov, Kirill A. Litvin, Iurii Iu. Grishin, Nikolay K. Balabaev, Elena Yu. Kramarenko

**Affiliations:** 1 Faculty of Physics, Lomonosov Moscow State University, Moscow 119991, Russia; kurbatov@polly.phys.msu.ru (A.O.K.); litvin.ka19@physics.msu.ru (K.A.L.); grishinyy@my.msu.ru (I.I.G.); balabaevnk@gmail.com (N.K.B.); 2 Institute of Mathematical Problems of Biology RAS—The Branch of Keldysh Institute of Applied Mathematics of Russian Academy of Sciences, Pushchino 142290, Moscow Region, Russia

**Keywords:** dendrimers, amphiphilicity, molecular dynamics, hydrogen bond

## Abstract

Amphiphilic dendrimers represent a promising class of nanoscale building blocks for functional materials, yet their conformational behavior, solvation, and interfacial activity remain incompletely understood. In this work, we employ atomistic molecular dynamics simulations to investigate G2–G4 carbosilane dendrimers functionalized with ethylene glycol terminal groups of two lengths—R1 (one ethylene glycol unit) and R3 (three units)—in water, toluene, and at fluid interfaces (water–toluene and water–air). Both types of dendrimers adopt compact, nearly spherical conformations in water but swell significantly (~83% in volume for G4) in toluene, a good solvent for the hydrophobic core. At the water–toluene interface, the dendrimers remain fully solvated in the toluene phase and show no surface activity. In contrast, at the water–air interface, they adsorb and adopt a mildly anisotropic, biconvex conformation, with a modest deformation. The total number of hydrogen bonds is reduced by ~50% compared to bulk water. Notably, the R3 dendrimers form more hydrogen bonds overall due to their higher oxygen content, which may contribute to the enhanced stability of their monolayers observed experimentally. These results demonstrate how dendrimer generation as well as terminal group length and hydrophilicity finely tune dendrimer conformation, hydration, and interfacial behavior, which are key factors for applications in nanocarriers, interfacial engineering, and self-assembled materials. The validated simulation protocol provides a robust foundation for future studies of multi-dendrimer systems and monolayer formation.

## 1. Introduction

Dendrimers are monodispersed, hyperbranched macromolecules with a regular tree-like architecture that confers a high density of functional groups on their surface [1,2,3,4]. Their distinct core–shell structure, in which the peripheral shell accounts for at least half of the molecular mass, makes them ideal platforms for tailoring intermolecular interactions and macroscopic behavior through surface chemistry and internal packing density [5,6,7].

Amphiphilic dendrimers are a particularly active research area [8]. They combine the intrinsic features of the dendritic scaffold, such as structural precision, defect-free geometry, host–guest encapsulation capability, and tunable surface functionality, with the self-assembly properties imparted by lyophilic (e.g., hydrophilic) surface groups. When properly functionalized, amphiphilic dendrimers can form stable Langmuir monolayers at fluid interfaces [8,9,10], dissolve in aqueous media as “monomolecular micelles,” [11] and self-assemble into aggregates of various morphologies [12,13,14]. These properties make them promising for drug delivery [15,16,17], sensing [18,19,20], and catalysis [21,22].

Among dendrimer families, carbosilane-based systems with a hydrophobic Si–C backbone and no strong directional interactions like hydrogen bonding in the core, serve as ideal model platforms to study how dendritic architecture affects macromolecular behavior in different environments [4,6,7,10,23,24,25,26,27,28]. Extensive experimental work has revealed remarkable generation-dependent phenomena in polybutylcarbosilane dendrimer melts, including a sharp liquid-to-solid transition between consecutive generations (e.g., from G5 to G6) with a ~10^6^-fold viscosity increase [24], and even crystalline ordering in melts of high-generation dendrimers [27,28]. Atomistic molecular dynamics (MD) simulations have further elucidated their local structure [29,30], viscoelastic response [31], and conformational changes of single molecules upon interfacial adsorption or compression [32,33].

Surface activity was first demonstrated for hydroxyl-terminated generations 0–4, which dissolve in alcohols [34] and form monolayers on hydrophilic substrates but aggregate on hydrophobic ones [35,36]. Higher generations suffer from steric crowding, reducing film quality due to limited conformational flexibility [37]. Atomistic MD simulations of OH-terminated carbosilane dendrimers (G2–G4) further linked molecular density to solvent uptake, hydrogen bonding, and interfacial restructuring in water, toluene, and at fluid interfaces [38,39].

More recently, ethylene glycol (EG)-functionalized carbosilane dendrimers (G1-G3) with a focal phenyl group were developed for biomedical applications, showing generation-dependent drug encapsulation [40,41]. Crucially, Langmuir monolayer studies of G3 and G6 dendrimers with mono- and tri- (ethylene oxide) terminals [10] demonstrated that interfacial stability increases with both generation and hydrophilic shell thickness, while higher rigidity promotes intermolecular ordering. However, a molecular-level understanding of how dendrimer generation and EG chain length jointly control solvation, conformation, and interfacial orientation remains lacking.

In the present work, we use atomistic MD simulations to study second- to fourth-generation carbosilane dendrimers functionalized with EG terminal groups of two lengths, in hydrophilic (water) and hydrophobic (toluene) solvents, and at water–toluene and water–air interfaces. By varying dendrimer generation (which controls core size) and terminal group length (which modulates hydrophilic shell thickness), we clarify how these structural parameters affect solvation, conformational flexibility, hydrogen bonding, and interfacial activity. Our results provide atomic-level explanations for the experimental trends in [10] and offer a rational basis for designing carbosilane-based amphiphiles with tailored functionality.

## 2. Materials and Methods

**Figure 1:**
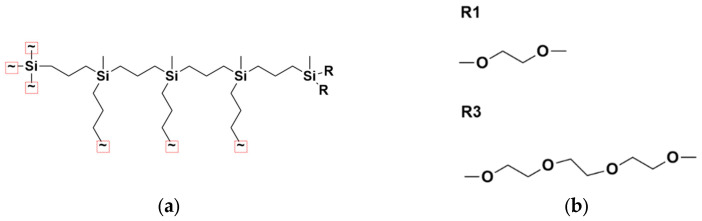
(**a**) Schematic representation of a fourth-generation dendrimer. (**b**) Chemical structures of the terminal group modifications: R = R1 (one EG unit) and R = R3 (three EG units).

We investigated the behavior of G2–G4 carbosilane dendrimers functionalized with ethylene glycol-based terminal segments. Figure 1a shows a schematic representation of the G4 dendrimer architecture. The dendrimers possess a four-functional Si core and three-functional branching points, namely =Si(CH3)-. Two variants were modeled, differing in the length of their terminal groups: –(OCH_2_CH_2_)_n_OCH_3_, with n = 1 and n = 3 (Figure 1b). For clarity, these are referred to herein as R1 and R3, corresponding to the “shorter” and “longer” terminal segments, respectively. The dendrimer composition is presented in Table 1.

All simulations were performed using molecular dynamics as implemented in GROMACS version 2021.1 [42,43,44,45]. A constant temperature of 298.15 K was maintained using the v-rescale thermostat [46] with a coupling time constant of 1 ps, while the pressure was kept at 1 atm using the Berendsen barostat [47] (time constant = 10 ps; compressibility = 4.5 × 10^−5^ bar^−1^). Dendrimers were modeled at full atomic resolution. Bonded parameters (bonds, angles, dihedrals) were taken from the AMBER force field [48,49], which has demonstrated good performance for organosilicon compounds and flexible alkyl/ether chains in condensed-phase simulations. The parameters of these potentials can be found in Ref. [38]. Partial atomic charges were assigned according to the PCFF force field [50], and long-range electrostatics were computed using the Particle Mesh Ewald (PME) method [51] with a real-space tolerance of 10^−5^. This hybrid AMBER/PCFF charge scheme was previously shown to reproduce the dendrimer sizes observed in experiments and experimental trends in dendrimer solubility and interfacial behavior, and was therefore adopted in the present work. Initial dendrimer conformations were generated following the standard protocol described in [29].

Each dendrimer was simulated in two homogeneous solvents (toluene and water) as well as at two distinct interfaces: water–toluene and water–vacuum. Toluene molecules were modeled using the AMBER force field, while water was described by the three-point flexible SPC/Fw model [52]. For homogeneous solvent simulations, vacuum-equilibrated dendrimer conformations were placed in a cubic simulation box (7 nm per side for G2 and G3 systems and 8 nm per side for the G4 system). The simulation box was sufficiently large to prevent self-interaction via periodic boundary conditions. The box was then solvated, and the system was equilibrated in the NPT ensemble.

For interfacial simulations, the initial configuration was constructed such that the dendrimer’s center of mass was positioned exactly at the interface. The simulation box featured two planar interfaces parallel to the XY plane, with the *Z*-axis perpendicular to them, and the aqueous layer had a thickness of 4 nm. Based on literature indicating that these dendrimer variants are soluble in toluene but insoluble in water [10], we placed two dendrimers—one at each water/air interface—to enhance conformational sampling. In contrast, only a single dendrimer was used in the water–toluene system to avoid intermolecular interactions within the toluene phase.

Simulations of G2 and G3 dendrimers were performed in the NVT ensemble at the both interfaces (water–air and water–toluene), using the v-rescale thermostat. Simulations of the G4 dendrimer at the water–air interface were also performed in the NVT ensemble, whereas those at the water–toluene interface used a semi-isotropic NPT ensemble, with pressure coupling applied only along the Z-direction (initial box length perpendicular to the interface was equal to 16 nm). Since our study focuses exclusively on equilibrium structural properties (e.g., density profiles, hydrogen bonding, conformational distributions) and not on dynamical or thermodynamic ensemble-sensitive quantities, the choice of ensemble does not affect our main conclusions. We have verified that structural observables (e.g., radial distribution functions, dendrimer shape parameters) are consistent between NPT (equilibration) and NVT (production) phases. Simulation trajectories for interface systems were extended up to 1 μs for G2 and for G3 and 500 ns for G4. To obtain reliable statistics for G4 interfacial systems, we adopted the following protocol. The dendrimer was first simulated at an elevated temperature of 600 K for 100 ns. At this temperature, the mobility of the terminal groups is significantly enhanced, and structural reorganization within the dendrimer occurs rapidly. Conformations sampled at regular intervals along this high-temperature equilibrium trajectory were then used as independent starting structures for relaxation at 298.15 K. Each conformation was relaxed for 100 ns, followed by a 200 ns production trajectory in the NVT ensemble used for time averaging. A total of 8 such independent realizations (with two dendrimers in the simulation box) were generated and combined for ensemble averaging. All reported averages for all systems were calculated from the equilibrated portions of the trajectories.

## 3. Results and Discussions

### 3.1. Dendrimer Size and Shape

**Figure 2:**
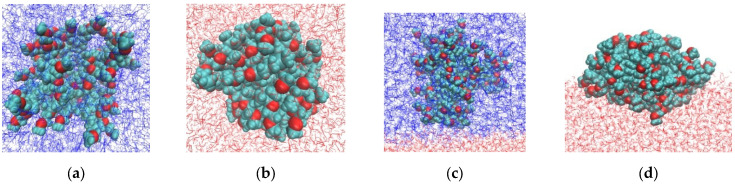

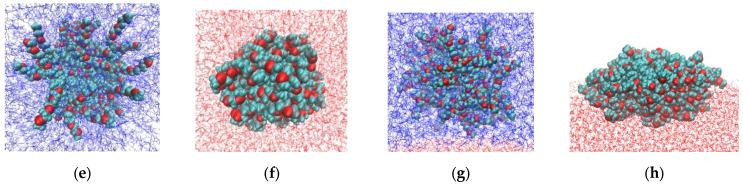
Snapshots of G4 dendrimers with short (R1, top row) and long (R3, bottom row) terminal segments in: (**a**,**e**) toluene, (**b**,**f**) water, (**c**,**g**) in the water–toluene system, and (**d**,**h**) at the water–air interface. Water and toluene molecules are shown in red and blue, respectively. Oxygen atoms in the dendrimer terminal segments are highlighted in red. Atoms are not drawn to scale.

Representative G4-system configurations are shown in Figure 2. The dendrimers appear more compact in water and more anisotropic at the water–air interface. At the water–toluene interface, dendrimers of both types do not exhibit surface activity and prefer to remain in the toluene phase. This result is consistent with the experimental data reported in [10] that the third and sixth generations of carbosilane dendrimers modified with EG terminal segments (R1 and R3) are insoluble in water. The observed surface activity at the water–air interface is also consistent with the experimental findings [10,41].

**Figure 3:**
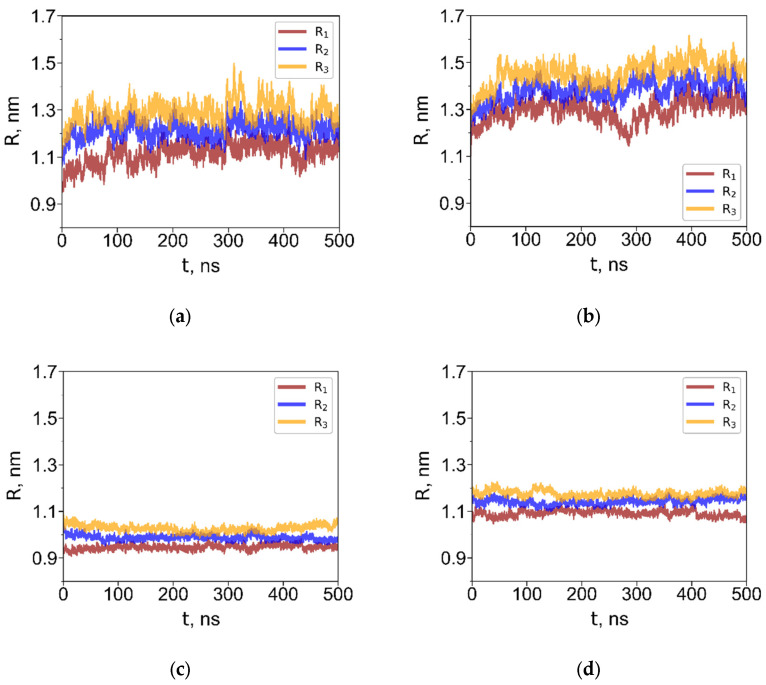

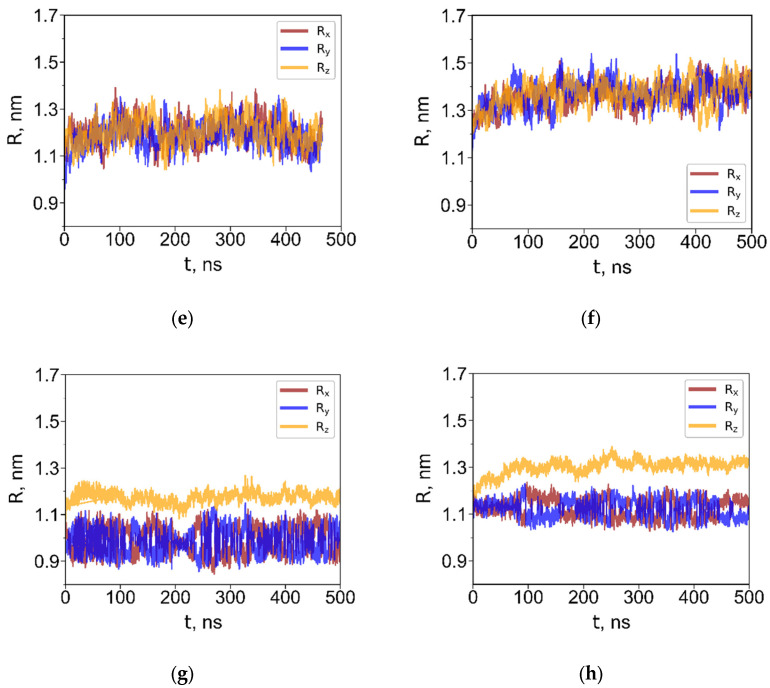
Radii of gyration about the principal axes (*R*_1_, *R*_2_, *R*_3_) for G4 dendrimers with the short (**a**,**c**) and long (**b**,**d**) terminal groups in toluene (**a**,**b**) and in water (**c**,**d**). Radii of gyration about the X-, Y- and Z-axes (*Rx*, *Ry*, *Rz*) for G4 dendrimers with the short (**e**,**g**) and long (**f**,**h**) terminal groups at the water–toluene interface (**e**,**f**), and at the water–air interface (**g**,**h**).

To characterize the size of the dendrimer depending on the solvent, we calculated the radii of gyration about the principal axes, denoted *R*_1_, *R*_2_, and *R*_3_ (Figure 3 shows their time evolution for G4 dendrimers). For interfacial systems, we calculated the radii of gyration about the X-, Y- and Z-axes, *Rx*, *Ry*, and *Rz* where the *Z*-axis is oriented perpendicular to the interface. One can see that *Ri* values rapidly reach equilibrium. The average equilibrium radii of gyration realized in homogeneous solutions are listed in Table 2.

In water, the dendrimers adopted compact, globular conformations with minimal size fluctuations. As expected, the R3 dendrimer exhibited a larger size than the R1 dendrimer due to its longer terminal groups. In toluene, which is a good solvent for the carbosilane core, the dendrimers swelled significantly. The degree of swelling of the largest dendrimers G4 in toluene, relative to their aqueous conformation, was approximately 83% in terms of volume and nearly identical for both dendrimer types. Notably, this value is almost twice as large as that reported for the G4 carbosilane dendrimer with hydroxyl terminal groups [38].

Since the dendrimers are not adsorbed at the water–toluene interface, the R components about the X-, Y- and Z-axes fluctuate and, on average, remain equivalent—interchanging values due to free rotational motion of the dendrimer in toluene. Consequently, the average radius of gyration is identical in pure toluene and in the water–toluene biphasic system. In contrast, adsorption at the water–air interface is characterized by a significant difference between the dendrimer dimensions along the *Z*-axis, $H_{z}$, and in the XY-plane, $H_{xy}$, indicating pronounced shape anisotropy. The values of the dendrimer dimensions along the X, Y, and Z axes were calculated as $H_{\alpha }=\sum_{i,j}{\left(\alpha_{i}-\alpha_{j}\right)}^{2}/2N$, where α=x,y,z; $\alpha_{i}$ and $\alpha_{j}$ being the corresponding coordinate projections of the *i*-th and *j*-th atom, $H_{xy}=\left(H_{x}+H_{y}\right)/2$. These values are shown in Table 2.

In each system, shape factors were calculated (see Table 3). In a homogeneous medium, these are defined as $K_{1}=\frac{R_{2}}{R_{1}}$ and $K_{2}=\frac{R_{3}}{R_{1}}$. The limiting cases correspond to distinct geometries: $K_{i}=1$ for a sphere, $K_{i}=0.5$ for a disk, and $K_{1}=0$, $K_{2}=1$ for an infinitely long thin rod. For a dendrimer at the interface, the shape factor is defined as $K=H_{xy}/H_{z}$.

The analysis shows that the dendrimers adopt a nearly spherical shape in homogeneous environments but exhibit a slight deformation at the water–air interface, spreading across the water surface. Compared to OH-functionalized dendrimers [38,39], the dendrimers studied here are even more spherical in solution owing to their longer terminal segments, and their interfacial deformation is less pronounced.

The higher the dendrimer generation, the less anisometric its shape at the water–air interface. At the interface, G2 dendrimers adopt a highly flattened conformation, with the lateral dimension approximately twice the perpendicular dimension. For the largest dendrimer, G4-R3, the value of K is reduced to approximately 1.6.

### 3.2. Solvent Penetration and Internal Dendrimer Structure

**Figure 4:**
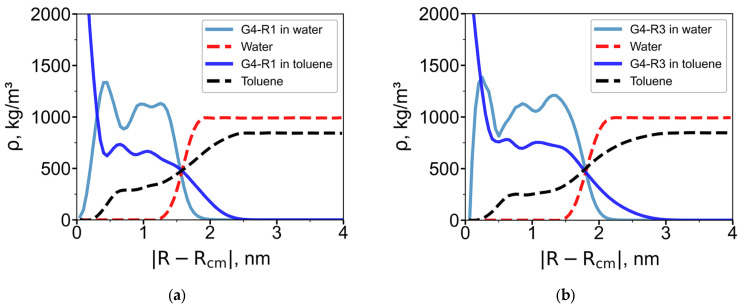
(**a**) Radial density profiles for systems containing (**a**) G4-R1 dendrimer and (**b**) G4-R3 dendrimer. The solid blue line represents the radial density profile of the dendrimer in toluene, while the dashed black line corresponds to toluene itself. Similarly, the solid light blue line shows the dendrimer in water, and the dashed red line represents water.

To assess solvent penetration into the dendrimer interior, radial density profiles of dendrimer and solvent around the dendrimer center of mass were calculated for systems in homogeneous solvents (Figure 4). They clearly show that both dendrimers do not absorb water while toluene can penetrate deeply into the dendrimer interior. The radial density profile for species *α* (*α* = d for dendrimer, *α* = w for water, and *α* = t for toluene) is defined as:

(1) $$\rho_{\alpha }\left(R\right)=\underset{i}{\sum }n_{i,\alpha }\left(R\right)m_{i}/V\left(R\right)$$

where $n_{i,\alpha }\left(R\right)$ is the number of atoms of type *i* belonging to species α in a spherical shell of volume $V\left(R\right)$ at radial distance *R* from the dendrimer’s center of mass, $m_{i}$ is the atomic mass, and the shell thickness is 0.5 Å.

Toluene, being a good solvent for the carbosilane core, penetrates deeply into the dendrimer interior. In contrast, water is largely excluded from the interior and is confined to the outer regions.

**Figure 5:**
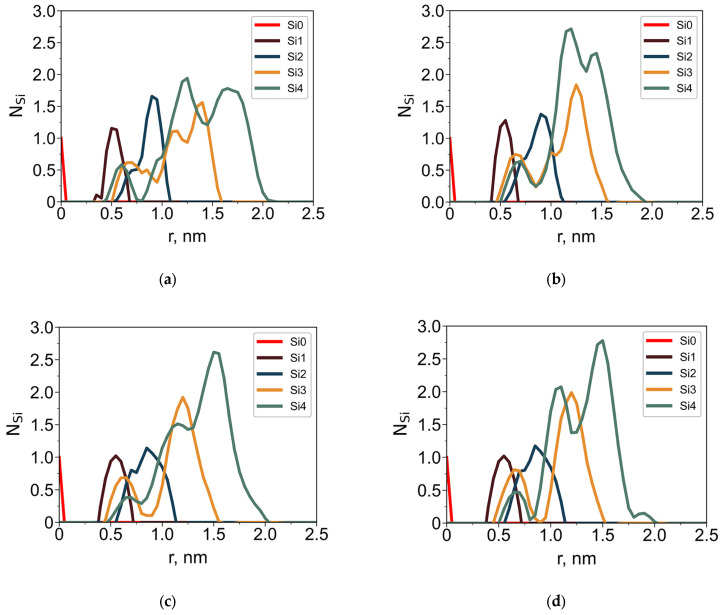
Average number of Si branching atoms, N_Si_, belonging to different structural layers as a function of the distance r from the dendrimer core Si for the G4-R1 (**a**,**b**) and G4-R3 (**c**,**d**) in toluene (**a**,**c**) and in water (**b**,**d**).

**Figure 6:**
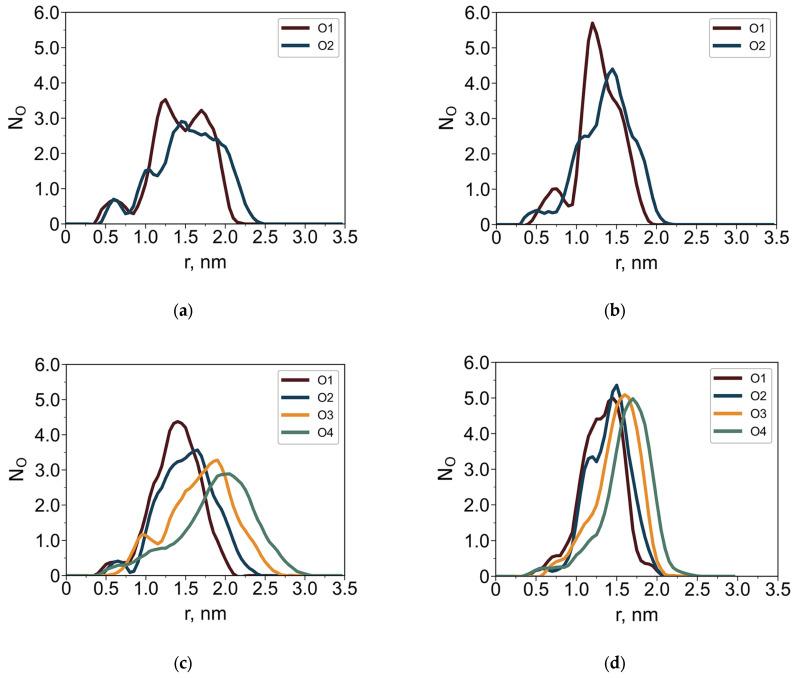
Average number of oxygen atoms, N_O_, as a function of the distance r from the core Si atom for dendrimers G4-R1 (**a**,**b**) and G4-R3 (**c**,**d**) in toluene (**a**,**c**) and in water (**b**,**d**). The oxygen atoms are numbered according to their structural position from the dendrimer core.

To further characterize the internal architecture of the dendrimers, radial distributions of silicon atoms (Figure 5) and oxygen atoms (Figure 6) of the terminal groups were analyzed separately. Structural layers were defined based on topological distance from the central silicon atom: each layer consists of Si atoms connected to the core by the same number of bonds (i.e., the layer number corresponds to the generation index). These layers are color-coded in Figure 5.

In both solvents, the dendrimers exhibit a distinct layered structure: the maxima corresponding to successive structural layers are well-resolved and shift outward with increasing layer number. Notably, silicon atoms from outer layers partially penetrate toward the core. This is a hallmark of backfolding, consistent with prior studies [29,38]. This phenomenon is observed in both toluene and water; however, key differences arise due to solvent–dendrimer interactions:

- In toluene, the dendrimer swells, causing all Si-layer peaks to shift to larger radial distances compared to water.
- In aqueous solution, the terminal groups move outward toward the periphery. This shift is driven by their hydrophilic nature, which favors contact with water.

The solvent-dependent behavior of the terminal groups is most evident in the oxygen distributions (Figure 6). In toluene, the oxygen distributions are broader than in water. Well-separated maxima corresponding to different oxygen atoms indicate a degree of elongation of the terminal groups in toluene. In contrast, in water the terminal groups adopt a more compact conformation and preferentially localize at the dendrimer periphery.

**Figure 7:**
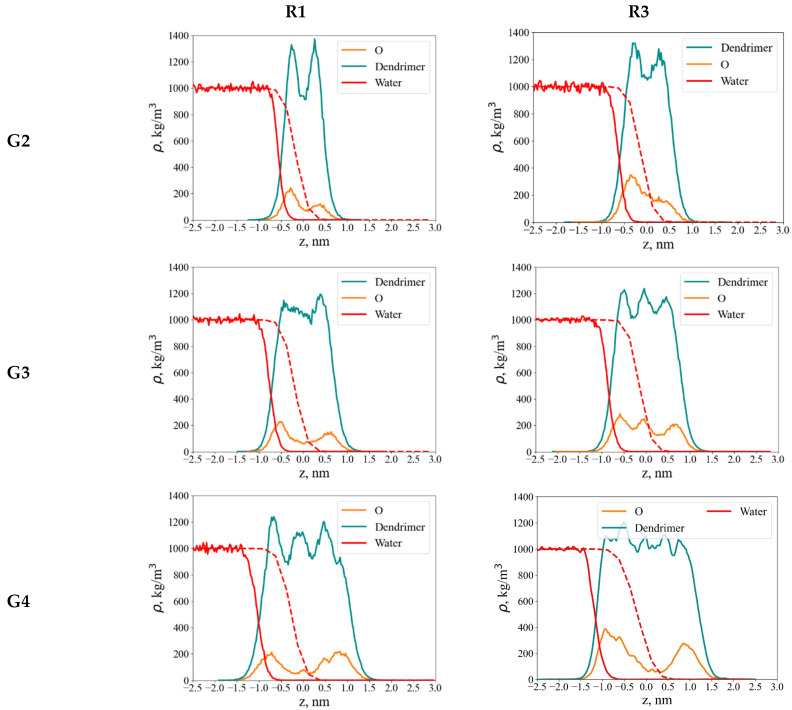
Axial density profiles along the *z*-axis at the water–air interface for G2–G4 dendrimers with R1 (left column) and R3 (right column) terminal segments: dendrimer monomer units, oxygen atoms, and water as indicated. The red dashed line shows the water density profile at the water–air interface in the absence of the dendrimer.

Because dendrimers adopt more anisotropic conformations at the interface, it is more informative to analyze their density distribution along the direction perpendicular to the interface. For both dendrimer types, axial density profiles were computed at the water–air interface within a cylinder of radius $H_{xy}$, oriented normal to the interface (Figure 7). The origin of the coordinate system coincides with the dendrimer’s center of mass.

In contrast to hydroxyl-terminated dendrimers, which adopt umbrella-like conformations at the water–air interface [39], those functionalized with less hydrophilic EG groups exhibit only minor conformational changes upon adsorption. These changes are clearly seen in some asymmetry of oxygen atom distributions for G2 and G3 dendrimers: O atoms more preferably interact with water. This asymmetry is less pronounced in the case of G4. Their axial density profiles show only a slight shift toward the aqueous phase. This finding is supported by both quantitative density profiles and visual inspection of dendrimer conformations (Figure 2). It contradicts the assumption in [10] that EG terminal segments lie flat (horizontally) on the water surface, emanating from the carbosilane globule. Our results demonstrate that they are mainly included into the dendrimer globule.

Water is largely excluded from the dendrimer interior. Nevertheless, because the dendrimer is partially immersed in the aqueous phase, its terminal oxygen atoms remain accessible to water molecules and can participate in hydrogen bonding.

### 3.3. Hydrogen Bonds

**Figure 8:**
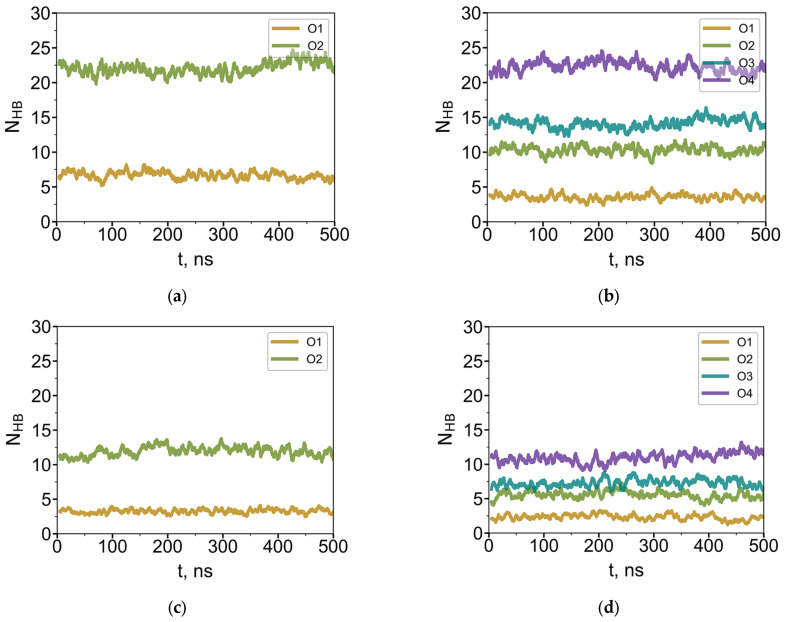
Current average number of hydrogen bonds (over 5 ns) between water molecules and oxygen atoms of G4-R1 (**a**,**c**) and G4-R3 (**b**,**d**) dendrimers in bulk water (**a**,**c**) and at the water–air interface (**c**,**d**).

Due to the absence of hydrogen bond donors within the dendrimer structure, time-dependent profiles of hydrogen bonds (HBs) formed by oxygen atoms in the terminal groups were calculated only for systems in which the dendrimer interacts with water (Figure 8). Hydrogen bonds were identified using the following geometric criteria: a HB was considered to be formed if (i) the distance between the donor and acceptor oxygen atoms was less than 0.35 nm, and (ii) the angle between the donor–hydrogen bond and the donor–acceptor vector was less than 30°. The average number of HBs formed by dendrimers of both types is presented in Table 4.

In each system, the number of hydrogen bonds per oxygen atom increases with distance from the dendrimer core (Figure 8 shows the time evolution of HB formed by each O-atom of G4-R1 and G4-R3). Oxygen atoms in the outermost layer are the most accessible for hydrogen bonding; both dendrimer types form the same number of HBs in this layer—22 ± 4. Given that the terminal O-layer of the G4 dendrimer contains 64 oxygen atoms, this corresponds to approximately one hydrogen bond per every third terminal oxygen. In contrast, oxygen atoms in the first (inner) layer form significantly fewer HBs, and this number depends on the length of the EG segment. Notably, these inner oxygens are slightly less accessible in G4-R3 than in G4-R1, yielding 4 ± 2 and 7 ± 2 HBs, respectively. Nevertheless, because G4-R3 contains more oxygen atoms overall, its total number of HBs with water is higher than that of G4-R1 (Table 4).

Table 4 compares the average number of hydrogen bonds formed by dendrimers with water molecules in aqueous solution and at the water–air interface. The number of hydrogen bonds is reduced by approximately half when the dendrimer adsorbs at the water–air interface. This is consistent with the dendrimer density profiles (Figure 7) and shape factor values (Table 3), which indicate only a modest conformational change upon adsorption, with roughly half of the dendrimer volume exposed to air in a biconvex conformation (see snapshots in Figure 2). The higher total number of hydrogen bonds formed by R3 compared to R1 may help explain the enhanced stability of monolayers formed by dendrimers with a thicker hydrophilic shell [10].

The findings for individual amphiphilic carbosilane dendrimers in solvents of varying polarity and at fluid interfaces lay the groundwork for ongoing investigations of many-particle systems, particularly dendrimer monolayers at the water–air interface. Moreover, this work is essential for deepening our understanding of amphiphilic dendrimer self-assembly and surface activity, and for guiding the design of novel dendrimer-based materials.

## 4. Conclusions

In this work, we employed atomistic molecular dynamics simulations to investigate the conformational behavior, solvation, and interfacial activity of G2–G4 carbosilane dendrimers functionalized with ethylene glycol terminal groups of two lengths: R1 (one EG unit) and R3 (three EG units), in homogeneous solvents (water and toluene) and at fluid interfaces (water–toluene and water–air).

Our results demonstrate that both dendrimer types adopt compact, nearly spherical conformations in water. In contrast, they swell significantly in toluene, which is a good solvent for the hydrophobic carbosilane core. The change in volume is approximately 83% for G4. This swelling is similar for both R1 and R3, even though their terminal segments have different lengths. It is also much greater than that seen in analogous hydroxyl-terminated dendrimers, highlighting the role of terminal group chemistry in modulating solvation.

At the water–toluene interface, the dendrimers show no surface activity and remain fully solvated in the toluene phase, as confirmed by their isotropic shape and unchanged radius of gyration relative to bulk toluene. This result has important implications for experimental monolayer formation. Effective Langmuir or liquid–liquid monolayers require strong interfacial anchoring, which is lacking here because the hydrophobic carbosilane core is well-solvated by toluene, while the short ethylene glycol termini are insufficiently polar to drive partitioning into the aqueous phase. Consequently, these dendrimers should exhibit weak surface activity. In contrast, at the water–air interface, both types of dendrimers adsorb and adopt a mildly anisotropic, biconvex conformation, with roughly half of their volume protruding into the air phase. Notably, this deformation is less pronounced than in OH-terminated analogs, tending to realize the maximum amount of hydrogen bonds with water molecules in umbrella-like conformations.

Analysis of branching atom distributions within the dendrimer interior reveals a well-defined layered structure with evidence of backfolding in both solvents. Toluene penetrates deeply into the dendrimer interior, whereas water is largely excluded, presenting only near the periphery where terminal oxygen atoms reside. Despite partial immersion at the water–air interface, these oxygens remain accessible for hydrogen bonding, though the total number of hydrogen bonds is reduced by ~50% compared to bulk water. This result is consistent with limited solvent exposure.

Hydrogen bonding analysis further shows that terminal oxygens in the outermost layer are the primary contributors to hydration. However, the longer R3 dendrimer forms more hydrogen bonds overall due to its higher oxygen count, even though its inner oxygens are slightly less accessible than those in R1.

Collectively, these findings illustrate how subtle modifications in terminal group length and chemistry can fine-tune dendrimer conformation, solvation, and interfacial behavior, which are key factors for applications in drug delivery, nanocarriers, and interfacial engineering. The methodology and force field combination employed here, validated against experimental data, provide a robust framework for future studies of functionalized dendrimers in complex environments.

## Figures and Tables

**Table 1:** Dendrimer compositions.

Dendrimer Type	Number of Dendrimer Atoms	Number of Oxygen Atoms
G2-R1	361	32
G2-R3	585	64
G3-R1	777	64
G3-R3	1225	128
G4-R1	1609	128
G4-R3	2505	256

**Table 2:** Radius of gyration of dendrimers in toluene and water, and in-plane (lateral) and out-of-plane (perpendicular) dimensions at the water–air interface.

Dendrimer Type	Water	Toluene	Water–Air Interface	
	*R_g_*, nm	*R_g_*, nm	*H_xy_*, nm	*H_z_*, nm
G2-R1	0.78 ± 0.02	0.93 ± 0.03	0.56 ± 0.03	0.30 ± 0.02
G2-R3	0.90 ± 0.02	1.18 ± 0.05	0.69 ± 0.03	0.32 ± 0.02
G3-R1	0.97 ± 0.01	1.21± 0.03	0.70 ± 0.03	0.38 ± 0.02
G3-R3	1.13 ± 0.02	1.48 ± 0.04	0.75 ± 0.03	0.41 ± 0.02
G4-R1	1.21 ± 0.01	1.48 ± 0.04	0.85 ± 0.03	0.51 ± 0.02
G4-R3	1.39 ± 0.01	1.70 ± 0.04	0.97 ± 0.03	0.59 ± 0.02

**Table 3:** Shape factors of dendrimers in toluene, water, and at water–air interface.

Dendrimer Type	Water		Toluene		Water–Air Interface
	$K_{1}$	$K_{2}$	$K_{1}$	$K_{2}$	K
G2-R1	0.92 ± 0.06	0.83 ± 0.05	0.90 ± 0.05	0.78 ± 0.04	1.90 ± 0.17
G2-R3	0.92 ± 0.07	0.80 ± 0.07	0.94 ± 0.05	0.82 ± 0.04	2.16 ± 0.18
G3-R1	0.91 ± 0.04	0.83 ± 0.04	0.94 ± 0.03	0.86 ± 0.03	1.82 ± 0.13
G3-R3	0.94 ± 0.05	0.87 ± 0.04	0.93 ± 0.04	0.85 ± 0.03	1.82 ± 0.12
G4-R1	0.92 ± 0.04	0.87 ± 0.04	0.96 ± 0.02	0.92 ± 0.02	1.66 ± 0.09
G4-R3	0.94 ± 0.03	0.89 ± 0.03	0.96 ± 0.02	0.93 ± 0.02	1.62 ± 0.07

**Table 4:** The average number/fraction of hydrogen bonds formed by dendrimers with water molecules.

Dendrimer Type	Water	Water–Air Interface
G2-R1	10 ± 3/0.31 ± 0.09	6 ± 2/0.19 ± 0.06
G2-R3	19 ± 3/0.30 ± 0.05	11 ± 2/0.17 ± 0.03
G3-R1	17 ± 3/0.27 ± 0.05	9 ± 2/0.14 ± 0.03
G3-R3	32 ± 5/0.25 ± 0.04	17 ± 3/0.13 ± 0.02
G4-R1	29 ± 4/0.23 ± 0.03	15 ± 2/0.12 ± 0.02
G4-R3	50 ± 6/0.20 ± 0.02	27 ± 3/0.10 ± 0.01

## Data Availability

The original contributions presented in this study are included in the article. Further inquiries can be directed to the corresponding author.
